# Supplementary material for: CMOS plus stochastic nanomagnets enabling heterogeneous computers for probabilistic inference and learning
Source: Nat Commun. 2024 Mar 27;15:2685. doi: 10.1038/s41467-024-46645-6 (PMC10973401; doi:10.1038/s41467-024-46645-6)
Supplement: Supplementary file 1 — Supplementary Information [file 41467_2024_46645_MOESM1_ESM.pdf]

SUPPLEMENTARY INFORMATION

I. BASIC PRINCIPLES OF PROBABILISTIC COMPUTING WITH HARDWARE P-BITS

Probabilistic algorithms (e.g., sampling, inference, optimization) are performed with a network of p-bits interacting with each other [1]. The basic equation for the p-bit is given by:

$$m_i(t+\tau_N) = \text{sgn}\{\text{rand}(-1,1) + \tanh[\beta I_i(t)]\}, \tag{1}$$

where rand(-1, 1) represents a random number drawn from the uniform distribution in $[-1,1]$, β is the inverse algorithmic temperature, and I_i is the local field of p-bit "i" received from its neighbors. τ_N in this equation is defined as the neuron evaluation time [2]. For the typical choice of 2-local (Ising-like) energy functions, I_i is given by:

$$I_i(t+\tau_S) = \sum J_{ij} m_j(t) + h_i, \tag{2}$$

where J_{ij} are the weights and h_i is the bias term for each individual p-bit. τ_S represents the synapse evaluation time. If the network of p-bits is symmetric ($J_{ij} = J_{ji}$), it is possible to define the following energy function:

$$E = -\left(\sum J_{ij} m_i m_j + \sum h_i m_i\right). \tag{3}$$

In such a network of N p-bits, there are 2^N states, $S = \{1, 2, \ldots, 2^N\}$, and each state $j \in S$ is visited according to the Boltzmann-Gibbs distribution:

$$p_j = \frac{1}{Z}\exp(-\beta E_j). \tag{4}$$

The coupled evolution of Supplementary Eq. (1) and Supplementary Eq. (2) represents a dynamical system and a wide variety of problems can be mapped onto this system, including combinatorial optimization, machine learning and quantum simulation problems, all phrased in terms of powerful physics-inspired Monte Carlo algorithms [3, 4]. In this paper, we focus on the two settings of probabilistic inference and Boltzmann learning. In either case, we will be interested in a fixed β value (typically 1) and sample from the corresponding Boltzmann-Gibbs distribution of the network. We will show how the asynchronous and truly random bits generated by the sMTJs represent a low-level realization of physics-inspired probabilistic computation.

II. CHARACTERIZING SMTJS THROUGH P-BITS

We first characterize the statistics of the sMTJs (in Supplementary Figure 1) by making measurements on the p-bit circuit described later in Supplementary Figure 2b. The fluctuations of the p-bit circuit are controlled entirely by the sMTJs. We observe the outputs of 5 sMTJ-based p-bits and characterize their rate of fluctuations from event times and autocorrelations.

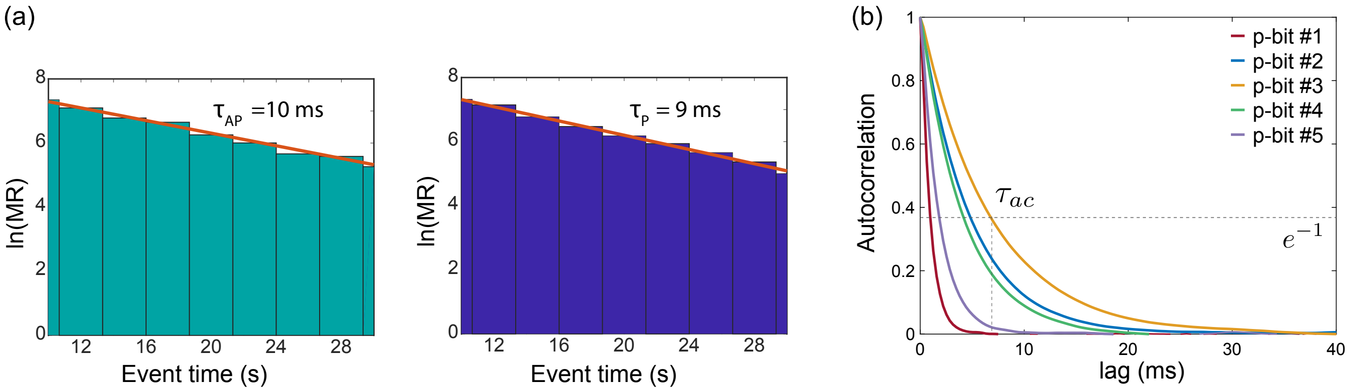

Supplementary Figure 1. sMTJ-based p-bit characterization. (a) Histogram of ln(MR) (number of magnetic relaxation (MR) events) as a function of the event times t_{event} for p-bit #2, following the exponential distribution $1/\tau_{\text{P,AP}} \exp(-t_{\text{event}}/\tau_{\text{P,AP}})$, expected from a Poisson process (red lines). (b) Autocorrelation of sMTJ-based p-bits #1-5. We define τ_{ac} as the time at which the normalized autocorrelation decays to $1/e$.

In the main paper FIG. 1g shows the fluctuations of p-bits #1-5 at $I_{50/50}$, which is the current at which the sMTJs show 50/50 fluctuations for the high- and low-resistance states. The rate of fluctuations provides an estimate of the neuron evaluation time τ_N of Supplementary Eq. (1) after which the p-bit produces a new and independent random bit.

p-bit	Mean MR time (τ)	Autocorr. time (τ_{ac})	TMR	$I_{50/50}$
1	2.4 ms	0.97 ms	64%	16 μA
2	9.6 ms	4.8 ms	68%	19 μA
3	14.4 ms	6.8 ms	65%	20 μA
4	8.7 ms	4.2 ms	59%	17 μA
5	4.2 ms	1.8 ms	65%	14 μA

Supplementary Table 1. Table of results for all 5 p-bits reporting mean relaxation time $\tau = \sqrt{\tau_{\mathrm{P}}\tau_{\mathrm{AP}}}$, τ_{ac}, TMR and $I_{50/50}$ of sMTJs. $I_{50/50}$ is defined as the absolute value of the current at which the perpendicular sMTJs show 50/50 fluctuations by canceling the uncompensated dipolar field resulting from the fixed layer.

Even though the sMTJs were fabricated under the same conditions, slight differences in their volumes and shapes critically affect their relaxation times. The relaxation times $\tau_{\mathrm{P,AP}}$ obeys a Néel-Arrhenius law [5], described by $\tau = \tau_0 \exp(\Delta/k_B T)$, where τ_0 is the attempt time and Δ is the energy barrier of the nanomagnet. In this paper, our magnets are not truly zero-barrier and their fluctuation rates exponentially depend on the energy barrier, Δ, however, detailed theoretical analysis shows that this dependence is much less pronounced in low barrier nanomagnets [6, 7]. Moreover, as we experimentally show in Sections 3-4, p-bits in symmetric networks are agnostic to update orders [2, 8, 9], therefore variations in these time scales should not play a prohibitive role in scaled implementations of p-computers (see Supplementary Note VIII for another experiment showing this update order invariance).

In Supplementary Figure 1b, we calculate the normalized autocorrelation of the p-bits using a 300 s time window with a sampling rate of 3.16 kHz, collecting $N \approx$ 949000 samples. The discrete autocorrelation function is defined as:

$$C[m] = \frac{\sum_{n=0}^{N-1} V[n]V[n+m]}{\sum_{n=0}^{N-1} V[n]^2}, \tag{5}$$

where $V[n]$ is the discrete sampled voltage read from the oscilloscope, and m represents the discretized lag time. Since autocorrelations typically show exponential decay [6], we extract an autocorrelation time τ_{ac}, by measuring and fitting the autocorrelation to a continuous function $C(t_{\mathrm{lag}}) = \exp(-t_{\mathrm{lag}}/\tau_{\mathrm{ac}})$. As an additional measure to τ_{ac}, we also determine the frequency of magnetic relaxations that are characterized by an event time. The event time, t_{event}, is defined as the time between one event to the next. Measuring the distribution of event times from parallel and antiparallel configurations, we observe that the p-bit outputs are distributed according to the exponential distribution, indicating a Poisson process [11]. Overall, p-bits show a good degree of variation in the relaxation and autocorrelation times as well as in their TMR values and their 50/50 currents. These values are summarized in Supplementary Table 1.

III. A VOLTAGE-COMPARATOR BASED P-BIT

In this note, we describe a new p-bit circuit we designed in this work, comparing its characteristics to an earlier design implemented in [10]. Supplementary Figure 2a,b show both these designs. The earlier design was implemented in several small-scale experimental demonstrations using perpendicular sMTJs [10, 12, 13]. In the original theoretical proposal [14], however, circular or elliptical in-plane nanomagnets were used. In-plane low barrier magnets are very hard to pin, requiring spin polarized currents of $\approx$100-500 μA or more for typical parameters [7]. If the magnetization provides continuous randomness providing all resistance values between R_{P} to R_{AP}, this allows a faithful realization of Supplementary Eq. (1) as carefully discussed in [15]. In such a case, spin-transfer-torque pinning is an unnecessary distraction, other than causing a read disturbance. Indeed, the fastest experimental p-bits are based on in-plane magnetic tunnel junctions [16, 17] and variations of the design proposed in Ref. [14] may still be useful in future implementations.

The experimental demonstrations including the present work have so far been primarily focused on perpendicular sMTJs to keep fluctuation speeds slow for practical reasons. Perpendicular sMTJs are easily pinned with spin currents around $\approx$10 to 20 μA for typical parameters [15]. Unlike in-plane sMTJs, however, perpendicular sMTJs switch telegraphically and they do not provide a uniform resistance between the two extremes. By a fortunate coincidence, the presence of the uncompensated dipolar field from the reference layer and easy pinning of perpendicular sMTJs appear to allow the realization of Supplementary Eq. (1) in hardware [10], since the spin-torque pinning changes the 50/50 fluctuations of the sMTJ [12].

In the design shown in Supplementary Figure 2a, the comparator has a fixed reference voltage. This means that as a function of V_{IN}, the drain voltage swings between two extremes, $V_{\mathrm{D,AP}} = V_{\mathrm{DD}} - R_{\mathrm{AP}} I_{\mathrm{MTJ}}$ and $V_{\mathrm{D,P}} = V_{\mathrm{DD}} - R_{\mathrm{P}} I_{\mathrm{MTJ}}$. As shown

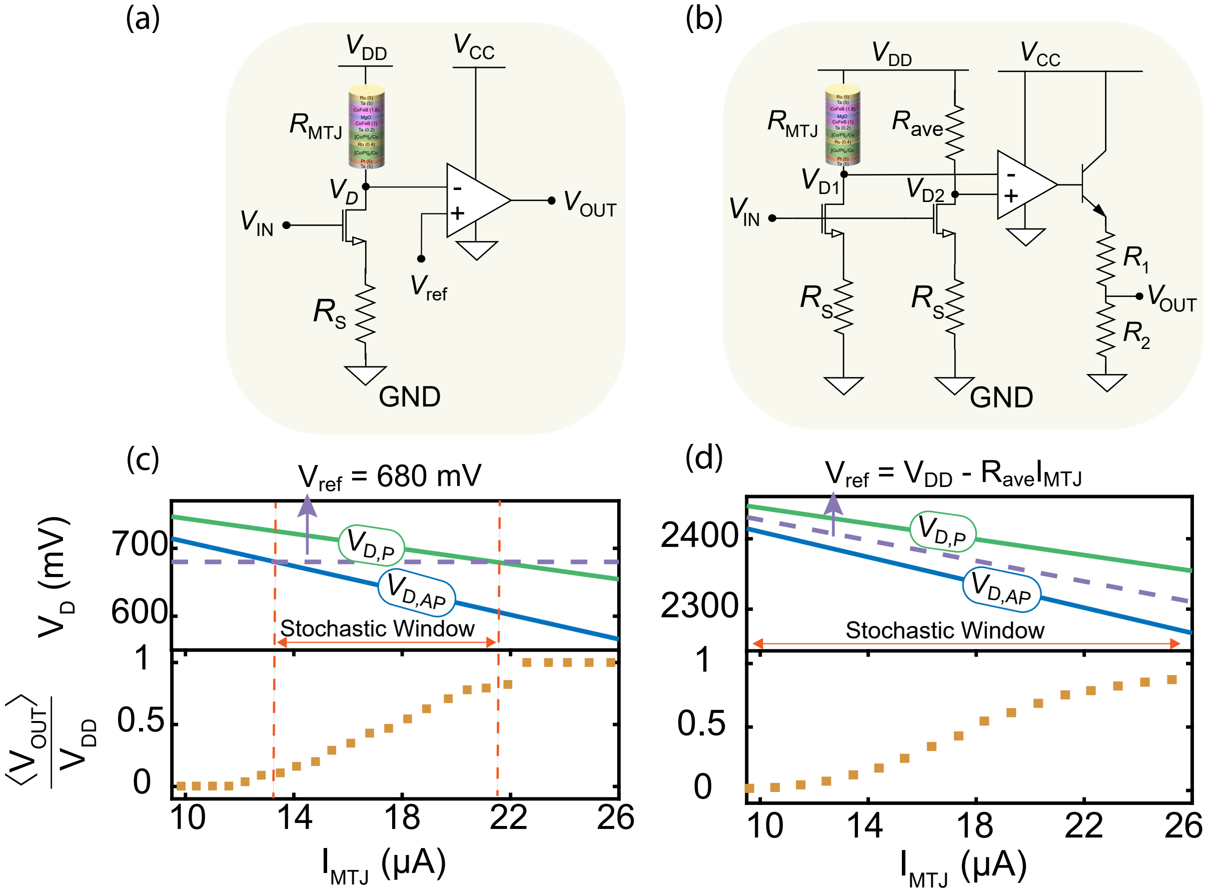

Supplementary Figure 2. sMTJ-based p-bit circuits (a) Single branch p-bit circuit diagram based on [10] with the following typical parameters: $V_{\mathrm{DD}} = 0.8$ V, $V_{\mathrm{CC}} = 2$ V, $R_{\mathrm{S}} = 10$ kΩ, $V_{\mathrm{ref}} = 680$ mV, and NMOS (2N7000) [10] (b) Double branch p-bit circuit with a variable V_{ref} and fixed R_{S}. $V_{\mathrm{DD}} = 2.5$ V, $V_{\mathrm{CC}} = 5$ V, $R_{\mathrm{S}} = 47$ kΩ, $R_{\mathrm{ave}} = 1/2\,(R_{\mathrm{P}}+R_{\mathrm{AP}})$, $R_1 = 100$ Ω, $R_2 = 220$ Ω, NMOS (ALD1101), and op-amp (ALD1702). (c) Single branch circuit of (a): the drain voltage as a function of current through the sMTJ, where ($V_{\mathrm{D,AP}} = V_{\mathrm{DD}} - R_{\mathrm{AP}} I_{\mathrm{MTJ}}$) and ($V_{\mathrm{D,P}} = V_{\mathrm{DD}} - R_{\mathrm{P}} I_{\mathrm{MTJ}}$). Vertical alignments show how the fixed reference voltage limits the stochastic window of the p-bit. (d) Double branch circuit of (b): the drain voltage as a function of the sMTJ current, showing the variable reference voltage to the op-amp.

in Supplementary Figure 2c, this limits the stochastic window of the p-bit. For the circuit shown in Supplementary Figure 2b, we have two parallel branches, one with the R_{MTJ} and the other with R_{ave}, defined as $1/2(R_{\mathrm{P}} + R_{\mathrm{AP}})$ of the sMTJ. The output nodes of these branches, taken from the drain of the transistor are compared by an operational amplifier. The key difference as shown in Supplementary Figure 2d is that the voltage reference is variable in the circuit of Supplementary Figure 2b and a larger stochastic window can be obtained. Additionally, the differential nature of the voltage comparison in the new design allows the use of a single source resistance across all sMTJs, unlike the circuit of Supplementary Figure 2a where the source resistance is adjusted individually [10]. Finally, the bipolar junction transistor at the output may not be necessary for integrated implementations, in this work, it simply functions as a buffer and lowers the output voltage to 3V, to safely interface with the FPGA.

IV. EXPERIMENTALLY DESIGNING THE VOLTAGE-COMPARATOR BASED P-BIT

Although the circuit shown in Supplementary Figure 2b is operated at only 50/50 fluctuations to provide asynchronous clocks, this circuit can also be used as a full p-bit whose output probability is tunable by the input voltage V_{IN}.

Each of the sMTJs used in this work has different characteristics, as such R_{ave} and V_{IN} should be customized for the specific sMTJ used in a p-bit circuit. We first characterize the specific sMTJ by experimentally measuring the $\langle R_{\mathrm{MTJ}} \rangle$ - I_{MTJ} (Supplementary Figure 3a), the value of R_{P}, and the value of R_{AP}. The value of the R_{ave} resistor is given by $R_{\mathrm{ave}} = 1/2\,(R_{\mathrm{P}} + R_{\mathrm{AP}})$.

As shown in Supplementary Figure 2b, the NMOS transistor and the R_{S} in either branch of the circuit form a constant

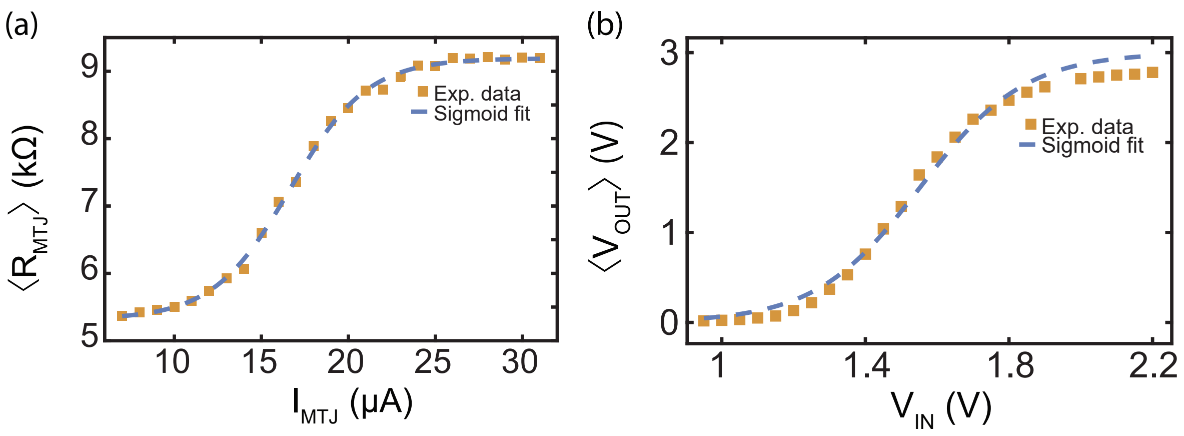

Supplementary Figure 3. sMTJ-based p-bit circuit characterization (a) $\langle R_{\mathrm{MTJ}} \rangle$, the time average (over a period of 3 minutes) of the sMTJ resistance, as a function of I_{MTJ}, the current flowing through the sMTJ. The yellow squares are experimental data, and the blue dashed line is a fit of the form $\langle R_{\mathrm{MTJ}} \rangle = a + b/\{1 + \exp\left[-c(I_{\mathrm{MTJ}} - d)\right]\}$, with $a = 5.32$ kΩ, $b = 3.87$ kΩ, $c = 0.45$ $(\mu\mathrm{A})^{-1}$, $d = 16.6$ μA. (b) Experimentally measured $\langle V_{\mathrm{OUT}} \rangle$, the time average (over a period of 3 minutes) of the output of the circuit shown in Supplementary Figure 2b, as a function of DC input voltage V_{IN}. The yellow squares are experimental data, and the blue dashed line is a fit of the form $\langle V_{\mathrm{OUT}} \rangle = 1/2\ V'_{\mathrm{CC}}[\tanh[\beta(V_{\mathrm{IN}} - V_0)] + 1]$, where $V_0 = 1.55$ V, $\beta = 3.43$ V^{-1}, $V'_{\mathrm{CC}} = 3$ V is a reduced voltage from $V_{\mathrm{CC}} = 5$ V.

current source. The two current sources in the two branches form a current mirror, so they have the same I-V characteristics. Note that the I-V characteristics of the circuit (with ALD1101 NMOS transistors and $R_{\mathrm{S}} = 47$ kΩ) do not depend on the R_{P} and R_{AP} values of the specific sMTJ used.

The ALD1101 current source can be characterized using the following setup: we replace the sMTJ and the R_{ave} in the left and right branches of the circuit with two 10 kΩ resistors, we connect V_{DD} to a 2.5 V power supply, and we leave V_{CC} unconnected. Supplementary Table 2 shows the experimentally measured I-V relationship.

V_{IN} (mV)	600	700	800	900	1000	1100	1200	1300	1400	1500	1600	1700	1800	1900	2000	2100	2200	2300	2400
I_{NMOS} (μA)	0.974	2.24	3.83	5.55	7.37	9.21	11.1	13.0	15.0	16.9	18.9	20.9	22.8	24.8	26.8	28.8	30.8	32.8	34.8

TABLE Supplementary Table 2. ALD1101 constant current source characterization: experimentally measured I_{NMOS} - V_{IN} relationship. The ALD1101 constant current source consists of the ALD1101 matched pair NMOS (the ALD1101 is a single IC consisting of two NMOS's with their threshhold voltages matched to within 10 mV of difference) and the two R_{S} resistors, as shown in Supplementary Figure 2b.

We discovered that the variation in the V_{TH} of each ALD1101 IC is important. While the shape of the I_{NMOS} - V_{IN} relationship in Supplementary Table 2 is the same for each ALD1101 IC, the V_{TH} variations lead to offsets, shifting the I_{NMOS} - V_{IN} curves up or down. Remeasuring of all the data points in Supplementary Table 2 is not required, only one measurement is needed: using the same characterization setup described above, we find and record the V_{IN} that produces $I_{\mathrm{NMOS}} = 16.9$ μA. The difference of this V_{IN} with the value recorded in Supplementary Table 2 (1500 mV) gives the offset to the entire I_{NMOS} - V_{IN} curve of the specific ALD1101 used. We simply add this offset to all data points in the reference I_{NMOS} - V_{IN} curve. We use the experimentally measured $\langle R_{\mathrm{MTJ}} \rangle$ - I_{MTJ} curve of the specific sMTJ and the I_{NMOS} - V_{IN} curve of the specific ALD1101 IC to determine the stochastic range of V_{IN}.

V. FPGA ARCHITECTURE

A. p-bit and MAC Unit

To evaluate the performance of the random number generators, we first implemented fully digital probabilistic bits (p-bits) on a Kintex UltraScale KU040 FPGA Development Board. A single p-bit consists of a tanh lookup table (LUT), a random number generator (RNG) and a comparator to implement Supplementary Eq. (1). In this work, we used 32-bit LUTs and RNGs. There is also a multiplier–accumulator (MAC) unit to compute Supplementary Eq. (2) from the neighbor p-bits and provide the input signal for the LUT. The p-bits are interconnected in a fixed hardware topology where the weights and biases, J_{ij}, h_i are stored in 10-bit registers and a digital multiply-accumulate operation with $m_j \in \{0, 1\}$ selects a particular weight or not. In the FPGA, we switch from a bipolar p-bit $m_i \in \{-1, 1\}$ to a binary formulation $m_i \in \{0, 1\}$ [18]. In our hybrid CMOS + sMTJ based p-bits, the architecture remains the same except the sMTJs serve as the clocks for the LFSRs and the p-bits.

B. RNG Unit

As shown in Algorithm 1 in the Supplementary information, we used three different types of RNGs: linear feedback shift register (LFSR), Xoshiro [19] and sMTJ-driven LFSR to compare the quality of randomness. We used 32-bit RNGs and compared the RNG outputs with the 32-bit LUTs to implement Supplementary Eq. (1). LFSR involves a linear shift operation on all the bits and an XNOR operation on some bits based on the selected taps. Unique seeds were used for each RNG and unique taps were used for RNGs in each p-bit block while ensuring maximal-length outputs. In contrast, a Xoshiro RNG involves linear shift and rotation operations on 32-bit words as well as XORing between subsequent words. In the all-digital versions, LFSR and Xoshiro RNGs are driven by digital clocks. However, in the hybrid design where sMTJs serve as the clocks for the RNGs, sMTJ + LFSR is considered as a new RNG unit. Using sMTJ-based p-bit removes the need for the 32-bit RNG and the 32-bit LUT.

C. Clocking and Sampling Unit

For the fully digital CMOS p-bits, each p-bit and its RNG is driven by system clocks generated on the low-voltage differential signaling (LVDS) clock-capable pins of the FPGA. These clocks are generated using Xilinx LogiCORE™ IP clocking wizard and mixed-mode clock manager (MMCM) module. Each of the five clocks operates at 15 MHz with shifted phases and is highly accurate with low jitter noise. In the Boltzmann learning example and for the NIST tests, to make these clocks comparable to sMTJs, we used frequency divider circuits to slow down the clocks to $\approx$ 2 kHz. For the sMTJ-driven p-bits, sMTJs replace the FPGA clocks and drive the p-bits and the RNGs externally using the peripheral module (PMOD) interface. FPGA samples the sMTJ outputs with a fast system clock of 75 MHz.

D. Data Programming and Acquisition Unit

We used MATLAB 2022a as the host program to read-write data to and from the FPGA through the USB-JTAG interface. MATLAB communicates with the FPGA board via AXI4 (Advanced eXtensible Interface 4) protocol where MATLAB works as the AXI master to drive a slave memory-mapped registered bank and Block RAMs (BRAM) inside the FPGA. We used airHDL [20], a memory management tool to assign the memory addresses for the register bank and the BRAMs. The weights J, h of the full adder circuit are programmed through MATLAB. In the inference and the Boltzmann learning example, the p-bit outputs were read from MATLAB as batches of sweeps that were initially sampled at 2kHz and stored in a BRAM. For the NIST tests, however, we sampled and stored all the sweeps in the BRAM at a much higher frequency ($\approx$10 kHz) compared to the clock frequency of $\approx$ 2 kHz and then downsampled the data to a designated frequency. This procedure ensured that we did not lose any samples. MATLAB reads the BRAM data in burst mode and performs the downsampling.

VI. INFERENCE ON THE PROBABILISTIC FULL ADDER

Inside the FPGA, we construct digital p-bits that behave according to Supplementary Eq. (1) and interconnections between p-bits that behave according to Supplementary Eq. (2). Each p-bit has a PRNG, a LUT for the hyperbolic tangent function and 10-bit weights in fixed-precision, 1 sign, 6 integer, and 3 fractional bits (s[6][3]). Supplementary Eq. (2) is implemented by a multiply-accumulate unit inside the FPGA, whose multiplication reduces to simple multiplexing since a given weight J_{ij} is either taken or not if m_j is 0 or 1. An important consideration in ensuring the p-bit network reaches the equilibrium is the necessity of fast synapse times (τ_s) compared to neuron times τ_n [2]. In our context this requirement ($\tau_s < \tau_n$) is naturally satisfied because the combinational logic inside the FPGA, which computes Supplementary Eq. (2) with about 10 ns delays is orders of magnitude faster than both our deliberately slowed digital clocks and our sMTJs. In scaled and integrated implementations with fast p-bits with GHz fluctuations, this necessity requires careful design. In the case sMTJ clocks, there is also the theoretical possibility of parallel updates by simultaneously switching sMTJs. Practically this is not a concern due to the extremely low probability of such an event which would be washed over thousands of samples anyway. Our results with sMTJs in FIG. 2c,d and in FIG. 3c,d indicate that the sMTJs reproduce the ideal distributions well.

A full block diagram of the FPGA unit is shown in FIG. 3a. To rule out any spurious correlations, the starting states (seeds) used for the LFSR and Xoshiro are randomized for each p-bit. LFSRs also use unique sets of random taps while ensuring maximum-length outputs.

In this setting, for each RNG, we cumulatively sampled 10^6 states from the p-bits starting from a random initial state. A system state out of 5-p-bits can be defined from 0 to $2^N - 1$ such that state 0 is $p_1p_2p_3p_4p_5 = 00000$ and state 31 is $p_1p_2p_3p_4p_5 = 11111$. We define the single update of each p-bit according to Supplementary Eq. (1)-(2) as a sweep. Due to their digital nature, defining exact times to perform a sweep for LFSR and Xoshiro is straightforward. With a driving clock frequency of 2 kHz, we perform one sweep and then record it as a new state. However, sampling states from sMTJ-clocked LFSR p-bits is not straightforward due to the analog nature of fluctuations of the sMTJs. The relaxation time of the slowest sMTJ is $\approx$20 ms (50Hz) that is 40$\times$ slower than the sampling frequency of 2 kHz. For this reason, we collect 40$\times$ more data points from sMTJ-based p-bits and downsampled them to obtain independent samples. The oversampling and subsequent downsampling of sMTJ data is due to our 40$\times$ faster readout process, not an inherent sMTJ limitation, and is implemented

to use the common sampler for each RNG setup. The sampling frequency can easily be adjusted, removing the need for oversampling and downsampling.

Algorithm 1: Learning the full adder on Deep BMs

```
Input  : number of samples N, number of truth table lines T, epochs N_L, learning rate ε, regularization λ
RNG: LFSR, Xoshiro, sMTJ-clocked LFSR
Output : learned weights J_new and biases h_new
J_new ← 0.01 * randi([−1, 1]);
h_new ← randi([−1, 1]);
for i ← 1 to N_L do
    J_FPGA ← J_new;
    /* positive phase                                   */
    for j ← 1 to T do
        h_T ← clamping to truth table values;
        h_FPGA ← h_T + h_new;
        {r} ← RNG();
        {m} ← FPGA(N, {r});
    end
    ⟨m_i m_j⟩_data = {m}{m}^T;
    /* negative phase                                   */
    h_FPGA ← h_new;
    {r} ← RNG();
    {m} ← FPGA(N × T, {r}) ;
    ⟨m_i m_j⟩_model = {m}{m}^T;
    /* update weights and biases                        */
    J_new ← J_new + ΔJ_ij;
    h_new ← h_new + Δh_i;
end
```

VII. LEARNING THE FULL ADDER ON THE 32-NODE CHIMERA LATTICE

Boltzmann machines can be trained using the contrastive divergence algorithm. There are two phases during the training of Boltzmann machines as shown in FIG. 3a and Algorithm 1. The first one is the positive phase where the network operates in its clamped condition under the direct influence of the training samples. The next one is the negative phase when the network is allowed to run freely without having any environmental input. The update rules can be obtained by minimizing the KL divergence between the data and the model distributions [21]:

$$\Delta J_{ij} = \varepsilon\Big(\langle m_i m_j\rangle_{\text{data}} - \langle m_i m_j\rangle_{\text{model}} - \lambda J_{ij}\Big) \tag{6}$$

$$\Delta h_i = \varepsilon\Big(\langle m_i\rangle_{\text{data}} - \langle m_i\rangle_{\text{model}} - \lambda h_i\Big), \tag{7}$$

where ϵ is the learning rate, λ is the regularization parameter, $\langle m_i m_j\rangle_{\text{data}}$ is the average correlation between two neurons in the positive phase, and $\langle m_i m_j\rangle_{\text{model}}$ is the average correlation between two neurons in the negative phase.

As described in the previous note, five sMTJ-based p-bits are used here as clocks for digital p-bits with 32-bit LFSRs in FPGA and each LFSR starts with a unique seed. We also used 5 different sets of taps for the LFSRs, ensuring maximal-length output. The $\langle m_i m_j\rangle_{\text{data}}$ is obtained by clamping visible bits to the eight lines in the truth table of the full adder (FIG. 2b). Then the $\langle m_i m_j\rangle_{\text{model}}$ is calculated in the negative phase where the clamp is removed. We obtain the updated weights and biases using Supplementary Eqs. (6),(7) and repeat the weight learning for 500 epochs. We also naturally adopt the persistent contrastive divergence (PCD) algorithm that runs a continuous Markov Chain from the last state of the previous update to the next [22]. This is because the FPGA holds the previous state of the chain and continues sampling from that state as new weights are loaded. The hyperparameters we used while learning are as follows: inverse temperature β = 1, learning rate ε = 0.003, and regularization λ = 0.005.

The 32-node Chimera graph is bipartite. This means that p-bits in a Chimera topology can be updated in parallel in two blocks. In order to make our system resemble eventual, fully-asynchronous systems, we distributed our 5 available sMTJ clocks over these two blocks, ensuring that no sMTJ clock serviced two p-bits of the same block to avoid parallel updates (FIG. 3b bottom panel shows our clock distribution over the p-bits). For the fully digital setup, we distributed the digital clocks similarly. As in the inference experiments in the previous note, LFSR and Xoshiro RNGs were driven at 2 kHz. We used the

same 5 sMTJ-based p-bits we characterized (Supplementary Figure 1) and sampled them at 2 kHz. To train the full adder on the LFSR and Xoshiro RNG-based p-bit network, we took 400 sweeps per epoch for a total of 500 epochs. For the sMTJ-clocked LFSR based p-bit network, we took 16000 sweeps per epoch and a total of 500 epochs. We took 40× more sweeps for the sMTJ because they were sampled at 2 kHz (40× faster than their autocorrelation) in order to produce the same number of independent sweeps for all solvers.

In Supplementary Figure 4 we provide the full histograms (32-states) of the full adder for the sampling and learning experiments. Only parts of the histograms are shown in the main text for clarity. In Supplementary Figure 8 we show that this bias is not random and never goes away, rather it is a consistent systematic bias by starting the LFSRs at different uniform random initial conditions (seeds) and different maximum-length taps.

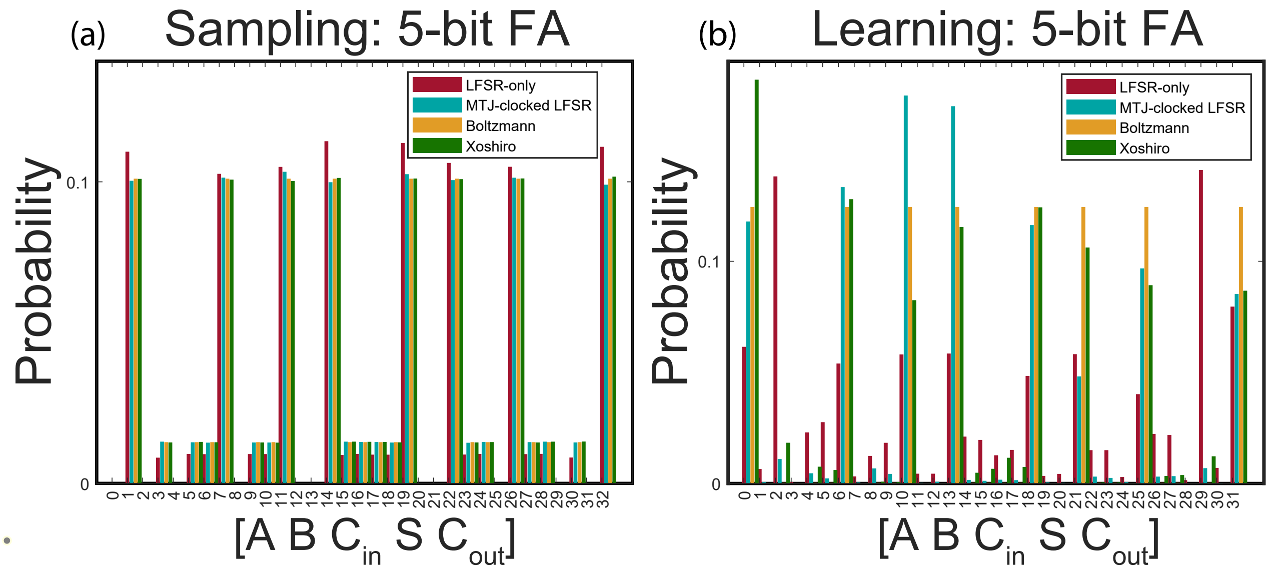

Supplementary Figure 4. Extended data for probabilistic sampling and learning experiments, showing all 32 states of the 5-bit full adder for LFSR, sMTJ-clocked LFSR, and Xoshiro. (a) Sampling data is taken at 10^6 sweeps, (b) Learning data is taken at epoch = 400 where the number of sweeps per epoch = 400 for LFSR-only and the number of sweeps per epoch = 16000 for the sMTJ-clocked LFSR.

VIII. UPDATE ORDER INVARIANCE OF BOLTZMANN NETWORKS

In Supplementary Figure 5, we perform additional experiments to study how undirected Boltzmann networks are invariant to update orders in the system, significantly easing experimental difficulties in scaled-out implementations of p-bit networks. In this experiment, a fully connected undirected p-bit network had nodes clocked asynchronously with independent sMTJs. Even with only 10^4 samples, a very close match is seen between the distributions corresponding to sMTJ-clocked LFSRs and Gibbs sampling in simulation. It is important to note that Gibbs sampling was performed with *randperm* updates, where a new update order (out of 4!=24 possibilities) was sampled (without replacement) at each iteration. Despite this highly random updating scheme, both Gibbs (ideal simulation) and the sMTJ-clocked system eventually reach the ground truth, represented by the Boltzmann distribution. This remarkable feature of update invariance in Boltzmann networks [8] significantly eases fabrication difficulties in eventual sMTJ-based large-scale p-bit networks. Going further, the investigation of "time-to-equilibrium" that determines the model averages and correlations in Algorithm 1 remains to be one of the future challenges of the field.

IX. SAMPLING FROM ARBITRARY DISTRIBUTIONS

In this note, we show how the CD algorithm can be used to create networks of p-bits that can sample from arbitrary distributions up to M-bit accuracy. We start by defining a PDF for a given distribution. As an example, we choose a normal distribution with $\mu = 16$ and $\sigma = 4$ to be approximated by a $N = 5$ p-bit network with $2^N = 32$ states. Once the PDF is specified, one approach is to create a truth table matrix of size $V = NT \times N$, where the rows of the truth table are repeated according to the specified PDF. Assuming a fully visible network, we then calculate the data correlations from the truth table, $V^{\dagger}V$. We then perform the standard contrastive divergence algorithm. Supplementary Figure 6 shows inference and learning of a normal and exponential distribution with Mersenne Twister-based high-quality RNGs and LFSR-based low-quality RNGs. Even though the Mersenne-based learning looks marginally better, we do not see a strong LFSR bias in such low-dimensional learning tasks. Next, we test the difference between low and high-quality RNGs in much larger networks including MNIST handwritten digits.

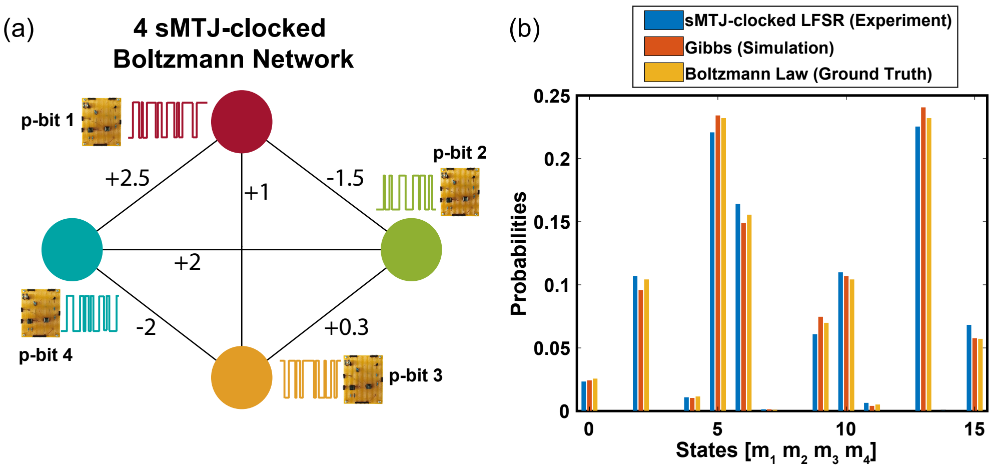

Supplementary Figure 5. Update order invariance of heterogeneous p-computer (a) Fully connected undirected Boltzmann network with 4 nodes. Different sMTJs drive the LFSRs in the p-bits corresponding to each node. (b) Experimental results of 4 sMTJ-clocked LFSR-based p-bits (1e4 iterations) performing probabilistic inference. This asynchronous clocking of LFSR-based p-bits matched the random Gibbs Sampling simulation results (1e4 iterations), which also matched the Boltzmann Law ground truth for these undirected networks. Note that the Gibbs simulation randomized the update order of the network, choosing one of 4!=24 possible permutations randomly at each iteration. The weight matrix is shown on the nodal interconnections, and the bias was 0 for p-bit 1, 3 and 4, but set to 0.3 for p-bit 2.

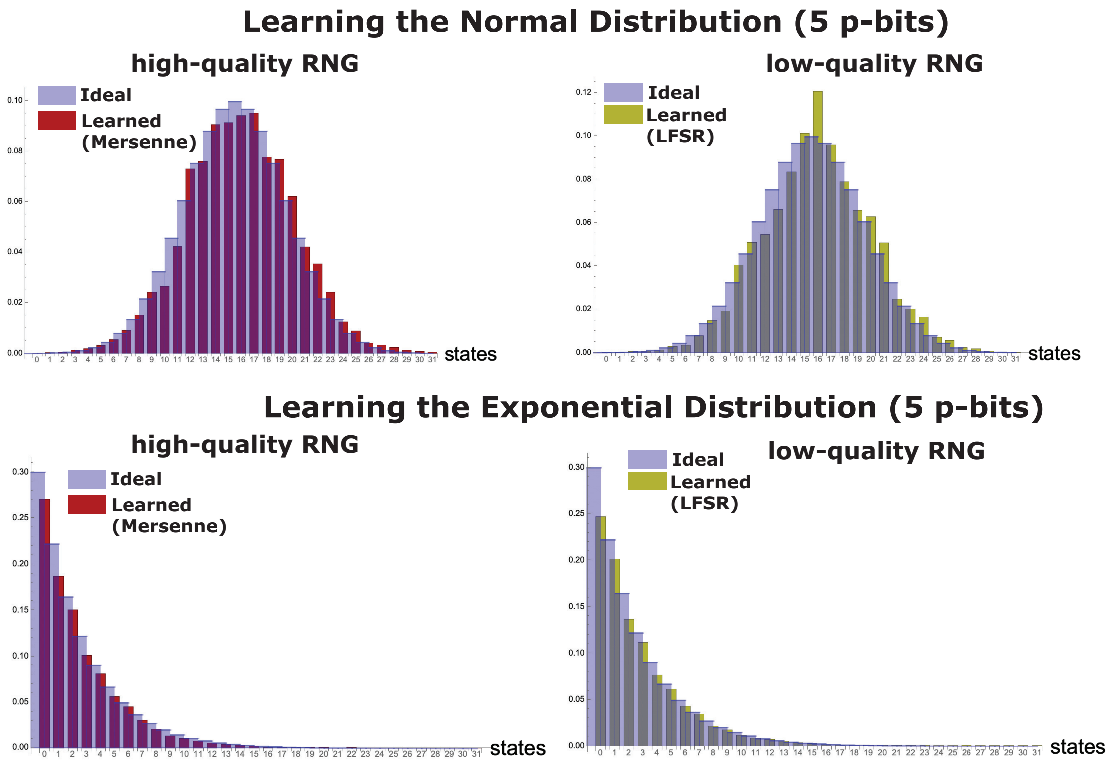

Supplementary Figure 6. Networks of p-bits can be connected to sample from arbitrary probability distributions: on the left of the top panel is a network of 5 p-bits approximating a normal distribution where training and inference are both done with high-quality Mersenne Twister-based PRNGs. The blue-filled lines are the exact probability density functions (PDF), discretely sampled in both figures. Bottom panel shows a similar result to learn the exponential distribution. Histograms show the inference on a 5 p-bit network that has been trained using the contrastive divergence algorithm. On the right is the same result (inference followed by learning) using 5 p-bits using LFSRs only (32-bit with random taps and initial conditions). All histograms are obtained with 50,000 samples. All models are trained on a 5-bit all-to-all fully visible network (weights and biases not shown).

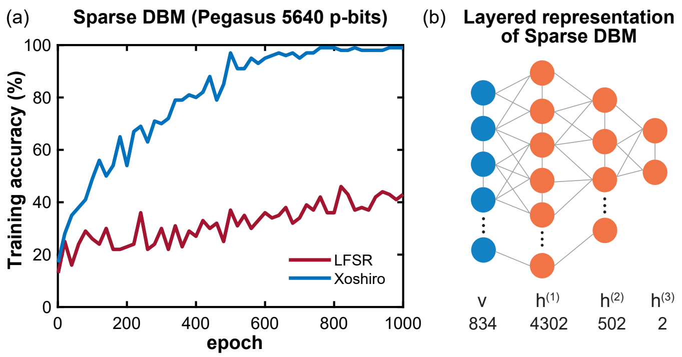

Supplementary Figure 7. Learning MNIST handwritten digits (a) Training accuracy of 100 handwritten digits from MNIST with a sparse Deep Boltzmann Machine (Pegasus 5640 p-bits) for 1000 epochs using both LFSR and Xoshiro. This shows a significant discrepancy in training accuracy depending on whether p-bits are implemented with LFSR or Xoshiro. Despite being identical excluding the RNG implementation, the former does not suffice due to the low accuracy at around 40% even after 1000 epochs, the latter reaches 100% accuracy with the same number of epochs and the same set of hyper-parameters. (b) Illustration of sparse DBM (Pegasus 5640 p-bits) with 1 layer of visible units and 3 layers of hidden units where both the inter-layer and intra-layer connections are allowed.

X. TRAINING MNIST WITH DEEP BOLTZMANN MACHINE: LFSR VS XOSHIRO

We now present a more complex task of training a subset of MNIST handwritten digits [23] using Algorithm 1 that also suffers from the low-quality randomness of LFSR. Pegasus 5640 p-bits [24] is chosen as the sparse DBM where we randomly distribute the visible and hidden units which yield multiple hidden layers as shown in Supplementary Figure 7b. The details of this method are described in [25]. Here, we choose a subset of MNIST data including 100 images with no down-sampling and train them in 10 mini batches using the CD algorithm for both LFSR and Xoshiro. All the hyperparameters remain the same for these two cases which are as follows: inverse temperature β = 1, learning rate ε varies linearly from 0.03 to 0.003 for 1000 epochs.

Supplementary Figure 7a shows the comparison of training accuracy of 100 MNIST digits between the implementation of LFSR and Xoshiro. While LFSR-based training barely reaches 40% accuracy in 1000 epochs, the Xoshiro-based training goes to 100% strongly indicating how the quality of randomness matters in terms of training such a large network.

XI. ANALYZING LFSR BIAS OVER DIFFERENT TAPS AND INITIAL CONDITIONS

Supplementary Figure 8 presents a detailed analysis of LFSR as a random number generator. LFSR RNG quality is measured by its ability to perform bias-free sampling on the probabilistic full adder. Across different seeds and taps, even for 10^6 iterations, a consistent bias is always seen as demonstrated by the variations in histogram peaks.

XII. NIST TESTS ON XOSHIRO, LFSR AND SMTJ CLOCKED LFSRS

NIST tests are widely used to evaluate the quality of randomness, so we conducted standard randomness tests on the bitstreams generated by the LFSR, Xoshiro, and LFSR+sMTJ using the NIST Statistical Test Suite [26]. We applied all 16 different NIST tests to the bitstreams. For LFSR and Xoshiro, we used MATLAB to generate bitstreams since given taps and initial conditions these bitstreams are fully reproducible.

We used a modified experimental setup to obtain the bitstreams for LFSR+sMTJ. First, the inverse temperature value β in Eq. (1) was set to 0 to get 50/50 fluctuations. Second, we sampled bitstreams of 650000 bits with a 10 kHz sampling rate in BRAM blocks of the FPGA. Then, we downsampled the bitstream by a factor k. This is equivalent to sampling the bitstream with frequency f_k that $f_k = 10000\ \mathrm{Hz}/k$. The length of the bitstream then becomes $650000/k$. The reason for this downsampling is to obtain independent samples from the sMTJs whose fluctuations times are far above 100 microseconds.

We used p-bit #3 to generate the bitstreams for LFSR+sMTJ. The result 'Random' represents that the bitstream passes the tests whereas 'Non-Random' means the bitstream fails. Supplementary Table 3 summarizes the results of the NIST tests for the LFSR+sMTJ with $k = 601$ and $k = 2001$, Xoshiro and LFSR. The results without oversampling issue show the good quality of randomness generated by LFSR+sMTJ when k=601 or $f_k = 16.63$ Hz, corresponding to a period of 60 milliseconds, of the same order of our sMTJ fluctuations reported in Supplementary Table 1. The result of $k = 2001$ fails Maurer's Universal Test (Test #9) because the downsampled bitstream with length $650,000/2001 \approx 324$ is not long enough for applying that test. Corroborating our results in the main text, LFSR+sMTJ and Xoshiro pass all the tests, while LFSR fails one test.

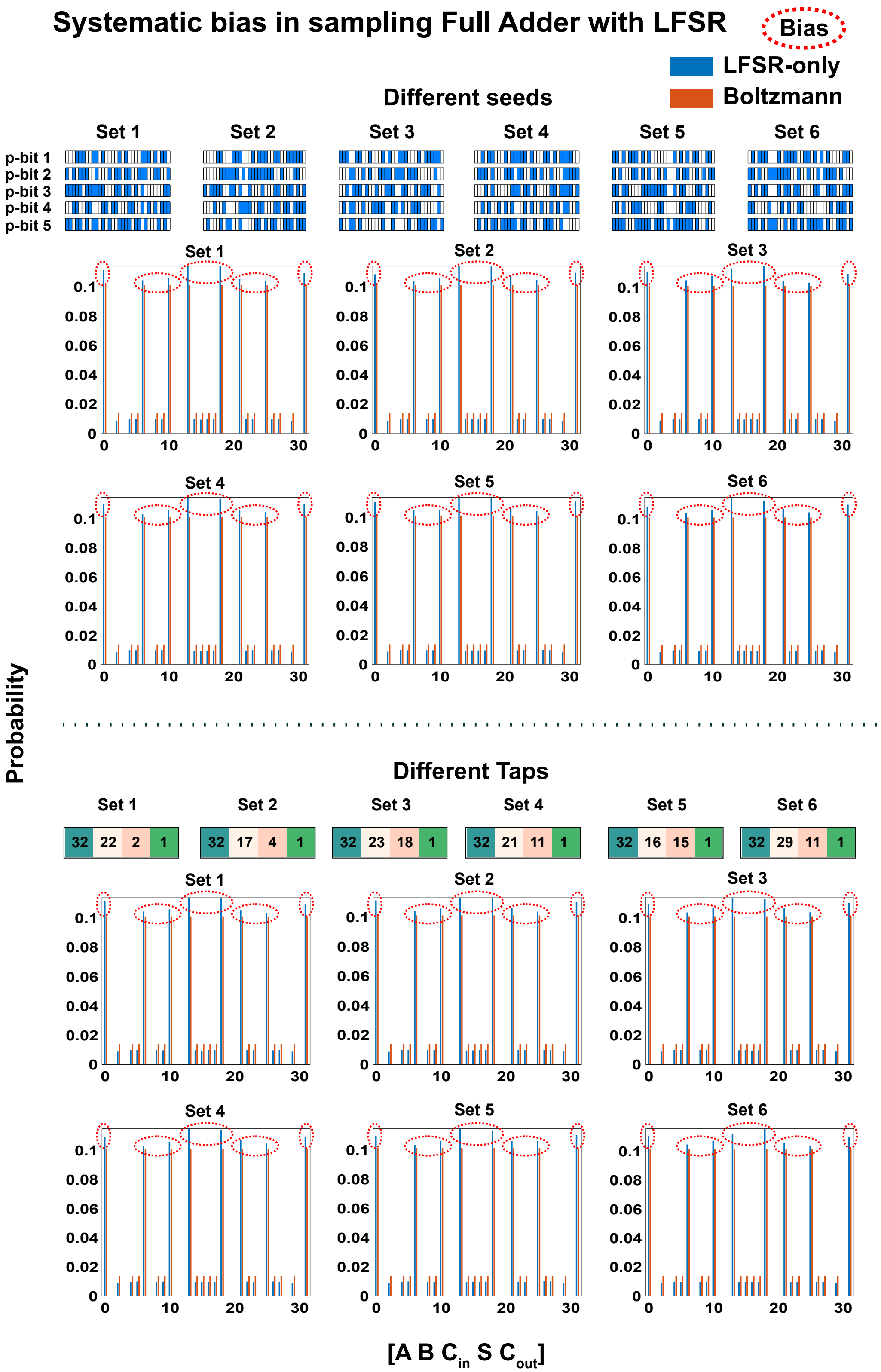

Supplementary Figure 8. Demonstration of consistent systematic bias in sampling the 5 p-bit full adder using multiple sets of seeds and taps for the LFSR. The seeds are chosen uniform randomly and the taps are chosen to provide the maximum length of the LFSR. Irrespective of the seeds and taps, the bias never goes away, indicating a systematic bias.

Supplementary Table 3. Results of tests on bit streams specified in standard NIST SP800-22a

Test #	Test Name	Sub-tests	LFSR + sMTJ $k = 601$ $f_k = 16.63$ Hz	LFSR+sMTJ $k = 2001$ $f_k = 5.00$ Hz	Xoshiro	LFSR
1	Frequency	1	Random	Random	Random	Random
2	Frequency within a Block	1	Random	Random	Random	Random
3	Runs	1	Random	Random	Random	Random
4	Longest run of ones	1	Random	Random	Random	Random
5	Rank	1	Random	Random	Random	Random
6	Discrete Fourier Transform	1	Random	Random	Random	Random
7	Non-overlapping T. M.	1	Random	Random	Random	Random
8	Overlapping T.M.	1	Random	Random	Random	Random
9	Maurer's Universal	1	Random	Non-Random	Random	Random
10	Linear complexity	1	Random	Random	Random	Non-Random
11	Serial	2	Random	Random	Random	Random
12	Approximate Entropy	1	Random	Random	Random	Random
13	Cumulative sums	2	Random	Random	Random	Random
14	Random Excursions	8	Random	Random	Random	Random
15	Random Excursions Variant	18	Random	Random	Random	Random

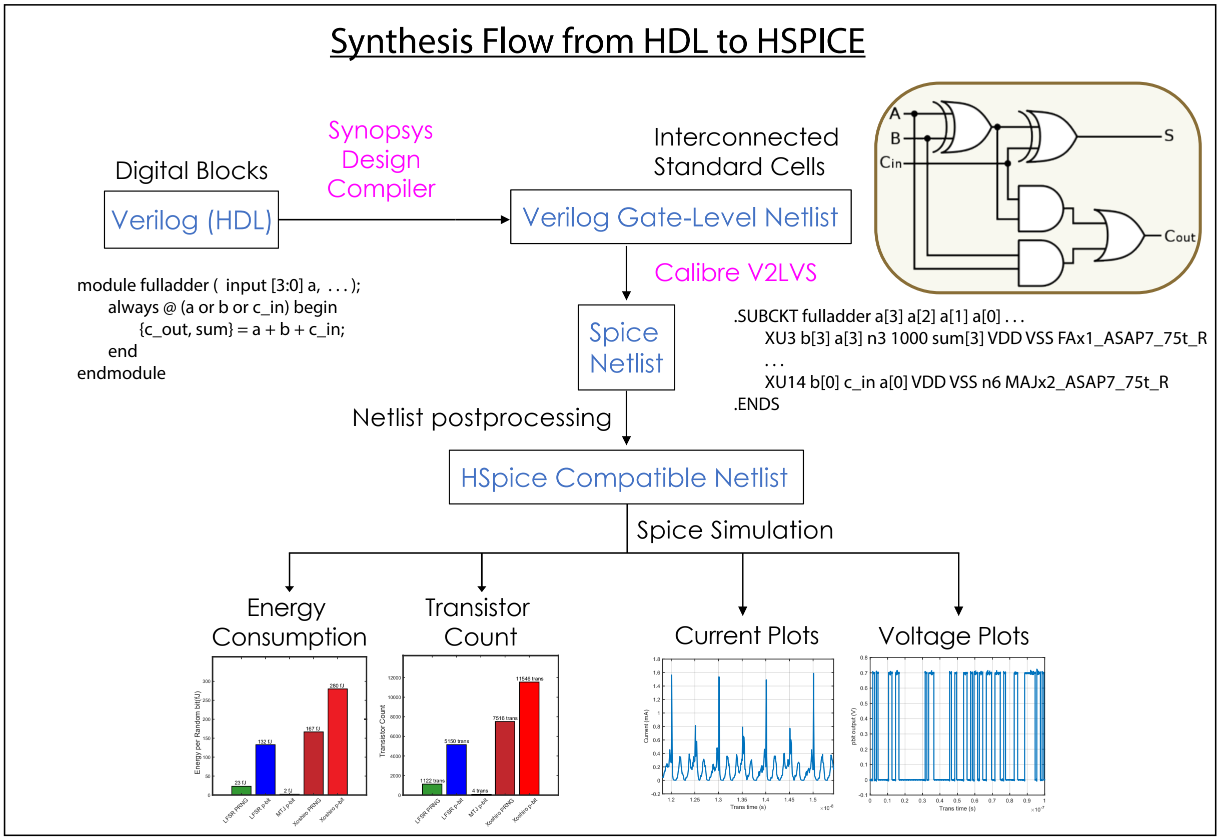

Supplementary Figure 9. Flowchart highlighting the key steps in the digital synthesis flow from hardware description languages such as Verilog all the way down to SPICE.

XIII. SYNTHESIS FLOW

The synthesis process followed involves the conversion of HDL codes modeling the digital p-bit to a functionally equivalent SPICE netlist (Supplementary Figure 9). This HDL-to-SPICE conversion first involves using Synopsys Design Compiler (DC) to generate the HDL gate-level netlist from the open-source ASAP7 PDK library files. Subsequently, we used Calibre's Verilog-to-LVS (V2LVS) tool to translate the gate-level netlist into a SPICE compatible netlist. After post-processing using a custom Mathematica script to get the netlist HSPICE compatible, we run transient simulations to obtain energy consumption, transistor counts, and current and voltage plots. With Synopsys DC, we used the regular voltage threshold (RVT) database files, and with V2LVS we used the RVT CDL files. For the HSPICE simulations, a VDD value of 0.7 V was used, and clock frequencies from

100 MHz to 1 GHz were tested, with 1 GHz being used for all results reported in this work.

XIV. FUNCTIONAL VERIFICATION OF P-BITS AND PRNGS

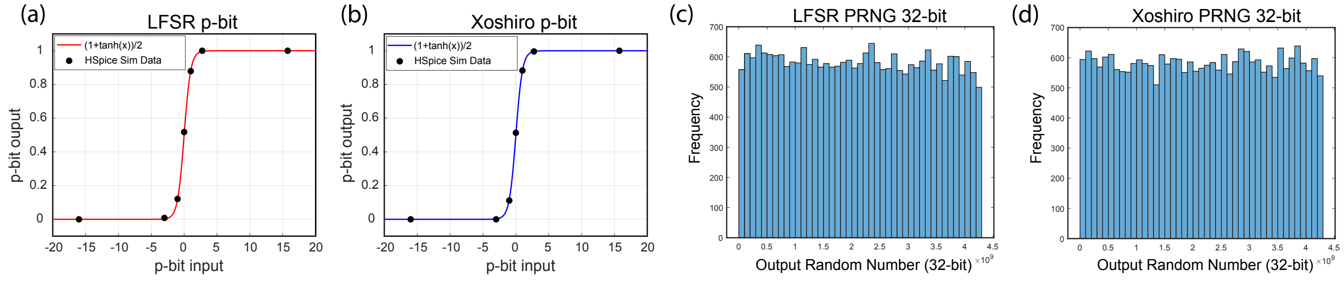

Supplementary Figure 10. Verifying p-bits and PRNGs functionality (a) 32-bit LFSR and (b) Xoshiro-based p-bits synthesized using ASAP7 PDK. Input current is converted from the 2's complement s43 representation to decimal equivalent. The y-axis indicates the probability of the p-bit being in a 1 state calculated over 2500 clock cycles of a transient p-bit simulation generating random bits. (c) Verifying the functionality of 32-bit LFSR and (d) Xoshiro PRNGs synthesized using ASAP7 PDK. 32-bit binary outputs were mapped to their decimal equivalent, and a 25000 clock cycle transient simulation generated random 32-bit values where $f_{\text{clk}} = 1$ GHz.

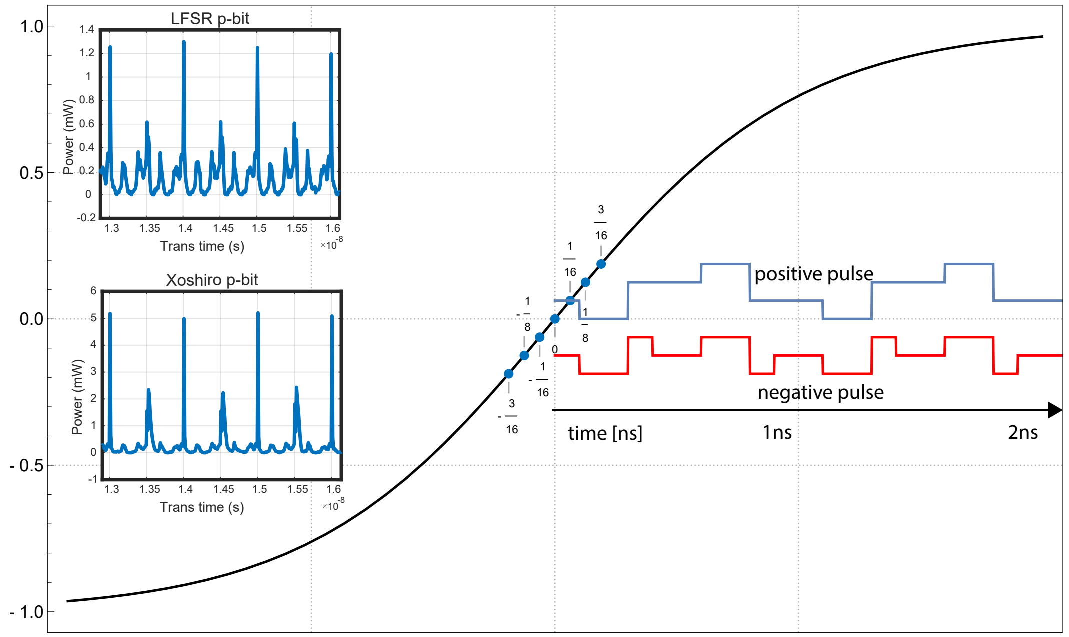

Supplementary Figure 11. Input profile to activate the LUT: 1 GHz positive and negative pulse are applied to the input of the p-bit to activate LUT transistors. Insets show representative transient simulations for instantaneous power consumption for LFSR and Xoshiro-based p-bits.

As seen from Supplementary Figure 10a,b, the experimental data obtained from HSPICE by varying the p-bit inputs (8-bit LUT input) of the synthesized circuit falls on the theoretically expected $1 + \tanh(x)/2$ for both LFSR and Xoshiro-based p-bits. Varying the p-bit input allows us to tune the probability with which the p-bit fluctuates in accordance with the sigmoid seen in Supplementary Figure 10a,b. In Supplementary Figure 10c,d, we observe that across 25000 experimental samples, the distribution of outputs obtained from the digitally synthesized PRNG (LFSR or Xoshiro) are uniformly random. These two results verify the functionality of the digital p-bit synthesized using ASAP7 by a transistor-level simulation performed in HSPICE.

XV. POWER ANALYSIS OF P-COMPUTERS

A. Synapse power

It is important to note that our calculations do not include any power analysis for the synapse (Supplementary Eq. (2)) so far. Earlier estimations [27] indicated that the synapse power is at least 50% of the overall power consumption. Depending on the implementation, for example, using analog crossbars or in-memory computing techniques, the synapse power could show a large degree of variation and we do not explore these possibilities in this paper.

B. Digital p-bit power

Insets of Supplementary Figure 11 show high resolution, representative section of the power plots for LFSR and Xoshiro p-bits. Energy analysis was performed by integrating the power over the transient time followed by averaging over 100 clock cycles of a 1 GHz clock, using trapezoidal numerical integration (trapz) in MATLAB.

In order to estimate the energy contribution of the LUT to the energy of generating a random bit, we vary the least significant 3 bits of the p-bit input to keep the LUT actively switching to simulate normal operating conditions during probabilistic computations. We do this in two different ways by generating 2 switching sequences of the form 00000xxx and 11111xxx, where "x" switches between 0 and 1 (see Supplementary Figure 11 for the pulse shapes). The first switching pattern is shown by the positive pulse in blue, where the LUT is traversing the sigmoid just above the zero point. The second switching pattern shown by the negative pulse in red has the LUT switching between values just below the zero point. We choose these input variations to have a 1 GHz frequency and measure the average power and energy dissipation over 100 clock cycles. For the positive pulse, the results are shown in FIG. 4b. For the negative pulse we obtain an energy consumption for a 32-bit LFSR p-bit as 119 fJ, and that of a 32-bit Xoshiro p-bit as 267 fJ, similar to what we observed for the positive pulse, reported in the main text, FIG. 4b.

XVI. BENCHMARKING AND PROJECTION OF P-BITS: ROADMAP

In this note, we provide projections and benchmarks for probabilistic inference and sampling hardware at device, circuit and systems levels which are summarized in Supplementary Table 4, below.

Device-level: At the sMTJ level, a key performance metric is the speed of fluctuations which can be measured by autocorrelation and magnetic relaxation times. Here, we seek order-of-magnitude estimates and represent average fluctuations by a single number τ for simplicity. There are two main methods to design sMTJs, one by employing nanomagnets with perpendicular anistropy (PMA) and another by employing nanomagnets with in-plane anisotropy (IMA). PMA magnets of the type we use in this paper typically have slow fluctuation rates compared to IMA [6, 28]. PMA magnets whose energy barriers are around 10-15 k_BT fluctuate in the millisecond range [10, 13]. On the other hand, recent device-level experiments using IMA magnets have shown fluctuations with 1-10 nanosecond rates [29–31]. sMTJs made out of IMA magnets possess other favorable properties such as bias-independence [16] to build robust p-bit circuits.

Circuit-level: Despite advances at the individual device level, p-bit circuits using sMTJs have not yet caught up with the fastest sMTJs. Even though we use a different current-mirror topology for the p-bit circuit in this paper, similar to earlier work, our p-bits use PMA MTJs with milisecond fluctuations. A recent report showed the fastest p-bit circuits with microsecond fluctuations, using IMA magnets [32]. Reaching nanosecond fluctuations with p-bit circuits might require integrated solutions, of the type that are sought initially in Ref. [33]. Given that CMOS circuitry could operate at picosecond timescales, there should be no fundamental obstacles to building GHz p-bit circuits.

System-level: At the system level, two main performance metrics for p-computers have been identified. One is the number of nodes in the network, N. The other one is sampling throughput that measures the number of probabilistic decisions taken by a system. Highly optimized standalone GPUs/TPUs of similar size graphs provide a sampling throughput of 10 flips/ns, establishing an optimized conventional baseline [34–37]. These chips consume around 100W of power. Given this GPU/TPU background, we provide established experimental data and projections for CMOS and CMOS+sMTJ-based probabilistic computers.

The FPGA columns represent our fully digital FPGA work with different quality RNGs (LFSR and Xoshiro). Depending on the bit precision and network connectivity, these digital solvers can sustain up to $N = 10^4$ p-bits in hardware [18, 25]. Running in parallel with around 10 MHz frequencies, they reach 100 flips/ns [18] in sampling throughput, about an order of magnitude higher than typical GPU and TPUs. On the other hand, the sMTJs with perpendicular magnetic anisotropy (PMA) used in our present heterogeneous p-computers currently have fluctuation times around $\tau = 1$ ms. However, near-term projections with $N = 10^4$ p-bits using sMTJs with in-plane magnetic anisotropy (IMA) ($\tau \approx$ 1ns [29–31]) can reach 10^4 flips/ns in sampling throughput. In the case of heterogeneous p-computers driven by sMTJs, the external p-bits can drive a large number of *digital* p-bits inside the FPGA. In such a case, around N=10,000 digital p-bits can be driven by sMTJs and the sampling throughput would be limited by the synapse time inside the FPGA (or the digital ASIC). This number shows that even in this modest scale, heterogeneous computers can already provide computational advantages over-optimized typical TPU/GPUs. Ultimate,

fully-integrated and sMTJ-based computers with $N = 10^6$ p-bits can reach sampling throughputs of 10^6 flips/ns, 5 orders of magnitude faster than typical TPU/GPUs. TPU/GPU references discussed have been plotted on a power consumption versus sampling throughput scale in [38] and [32].

For sampling throughput projections at large scale, we use N/τ, assuming each p-bit flips independently of each other. This basic formula assumes that each flip is communicated to neigboring p-bits *before* a new flip is attempted, as otherwise flips may not be useful. If the network density scales as $O(k \cdot N)$ with some small k corresponding to sparse networks, the fast communication assumption is warranted and ideal parallelism can be achieved.

For FPGA-based p-computers, the total power consumption is around 30W, including peripheral and unrelated circuits beyond the digital synthesis of our design. For heterogeneous computers of the type we consider in this work (5 MTJs + FPGA), the total power is also dominated by the FPGA power and is also around 30W. Unlike the present work where we used sMTJs to clock digital PRNGs such as LFSRs, in the future we envision the sMTJ-based analog p-bits as standalone blocks interacting through a CMOS underlayer without any seeding of CMOS PRNGs. In terms of power estimates for such fully sMTJ-based p-computers, detailed circuit simulations with experimentally established parameters indicate a power consumption of 10 μW per p-bit [15]. For fully sMTJ-based p-computers with $N = 10^6$, this would indicate a total p-bit power of 10 W, with an additional 10 W estimated synapse power [27, 32]. Considering how present day MRAM technology has been scaled up to 1 Gbit densities [39], integrating about 10^6 p-bits on top of CMOS should be reasonable.

Supplementary Table 4. Benchmarking probabilistic hardware from device, circuit and system perspectives. For device comparisons we focus on experimentally demonstrated sMTJ fluctuations. p-bits are circuits that use sMTJs to produce binary stochastic neurons with tunable probability with fluctuations at τ^{-1} rates. At the system level, we focus on number of p-bits in a network (N) and sampling throughput, which is given by N/τ, for asynchronous systems with fast synapses computing Supplementary Eq. 2 (see text). Also at the system level, we report published data for GPUs/TPUs handling similar probabilistic sampling tasks. Projections are shown using (†). (*) In heterogeneous computers of the type we consider in this paper, external sMTJ-based p-bits can drive a large number of digital p-bits in an FPGA or an ASIC. $^{\circ}$ RNG quality is deemed low / high for *sampling* problems rather than combinatorial optimization problems for which LFSR-based PRNGs seem sufficient [18].

		⟵ THIS WORK ⟶				NEAR-TERM PROJECTION		LONG-TERM PROJECTION
Level of Comparison	GPUs/TPUs	FPGA p-computer		Hetero FPGA+sMTJ p-computer			ASIC all-digital	Heterogeneous sMTJ+ASIC
		LFSR-based	Xoshiro-based	PMA	IMA	IMA		
**DEVICE:** sMTJ	N/A	N/A	N/A	$\tau \approx 1\ ms$ [10]	$\tau \approx 1\ \mu s$ [17]	$\tau \approx 1\ ns$ [30]	N/A	$\tau \approx 1\ ns$†
**CIRCUIT:** p-bit	N/A	$(\tau)^{-1} \approx 10\ MHz$	$(\tau)^{-1} \approx 10\ MHz$	$\tau \approx 1\ ms$ [10]	$\tau \approx 1\ \mu s$ [32]	$\tau \approx 1\ ns$†	$\tau \approx 1\ ns$†	$\tau \approx 1\ ns$†
**SYSTEM:** Number of p-bits (N)	N/A	$N \approx 10^4$ [18]	$N \approx 10^4$ [25]	$N = 32(*)$	$N \approx 10^4(*)$†	$N \approx 10^4(*)$†	$N \approx 10^5$†	$N \approx 10^6$†
**SYSTEM:** Sampling throughput (flips/ns)	≈ 10	≈ 100	≈ 100	$\approx 32 \times 10^{-6}$	≈ 10†	$\approx 10^4$†	$\approx 10^5$†	$\approx 10^6$†
RNG Quality	PRNG/ High	PRNG/ Low°	PRNG/ High	TRNG/ High	TRNG/ High	TRNG/ High	PRNG/ High	PRNG/ High

REFERENCES

[1] Kerem Y Camsari, Brian M Sutton, and Supriyo Datta. P-bits for probabilistic spin logic. *Applied Physics Reviews*, 6(1):011305, 2019.

[2] Rafatul Faria, Jan Kaiser, Kerem Y Camsari, and Supriyo Datta. Hardware design for autonomous bayesian networks. *Frontiers in Computational Neuroscience*, 15:584797, 2021.

[3] Jan Kaiser and Supriyo Datta. Probabilistic computing with p-bits. *Applied Physics Letters*, 119(15):150503, 2021.

[4] Shuvro Chowdhury et al. A full-stack view of probabilistic computing with p-bits: devices, architectures and algorithms. *IEEE Journal on Exploratory Solid-State Computational Devices and Circuits*, 9(1):1–11, 2023.

[5] William T Coffey and Yuri P Kalmykov. Thermal fluctuations of magnetic nanoparticles: Fifty years after brown. *Journal of Applied Physics*, 112(12):121301, 2012.

[6] Jan Kaiser et al. Subnanosecond fluctuations in low-barrier nanomagnets. *Physical Review Applied*, 12(5):054056, 2019.

[7] Orchi Hassan, Rafatul Faria, Kerem Yunus Camsari, Jonathan Z Sun, and Supriyo Datta. Low-barrier magnet design for efficient hardware binary stochastic neurons. *IEEE Magnetics Letters*, 10:1–5, 2019.

[8] Emile Aarts and Jan Korst. *Simulated annealing and Boltzmann machines: a stochastic approach to combinatorial optimization and neural computing*. John Wiley & Sons, Inc., 1989.

[9] K. Y. Camsari et al. Stochastic p-bits for invertible logic. *Physical Review X*, 7(3):031014, 2017.

[10] William A Borders et al. Integer factorization using stochastic magnetic tunnel junctions. *Nature*, 573:390–393, 2019.

[11] Takuya Funatsu, Shun Kanai, Jun'ichi Ieda, Shunsuke Fukami, and Hideo Ohno. Local bifurcation with spin-transfer torque in superparamagnetic tunnel junctions. *Nature communications*, 13(1):4079, 2022.

[12] Ahmed Zeeshan Pervaiz, Supriyo Datta, and Kerem Y Camsari. Probabilistic computing with binary stochastic neurons. In *2019 IEEE BiCMOS and Compound semiconductor Integrated Circuits and Technology Symposium (BCICTS)*, pages 1–6. IEEE, 2019.
[13] Jan Kaiser et al. Hardware-aware in situ learning based on stochastic magnetic tunnel junctions. *Physical Review Applied*, 17(1):014016, 2022.
[14] Kerem Yunus Camsari, Sayeef Salahuddin, and Supriyo Datta. Implementing p-bits with embedded mtj. *IEEE Electron Device Letters*, 38 (12):1767–1770, 2017.
[15] Orchi Hassan, Supriyo Datta, and Kerem Y. Camsari. Quantitative evaluation of hardware binary stochastic neurons. *Phys. Rev. Applied*, 15:064046, Jun 2021.
[16] Kerem Y Camsari et al. Double-free-layer magnetic tunnel junctions for probabilistic bits. *Phys. Rev. Appl.*, 15:044049, 2021.
[17] Keito Kobayashi et al. External-field-robust stochastic magnetic tunnel junctions using a free layer with synthetic antiferromagnetic coupling. *Physical Review Applied*, 18(5):054085, 2022.
[18] Navid Anjum Aadit et al. Massively parallel probabilistic computing with sparse ising machines. *Nature Electronics*, 5(7):460–468, 2022.
[19] David Blackman and Sebastiano Vigna. Scrambled linear pseudorandom number generators. *ACM Trans. Math. Softw.*, 47(4), 2021.
[20] airhdl.com. airhdl VHDL/SystemVerilog Register Generator. https://airhdl.com.
[21] David H Ackley, Geoffrey E Hinton, and Terrence J Sejnowski. A learning algorithm for boltzmann machines. *Cognitive science*, 9(1): 147–169, 1985.
[22] Asja Fischer and Christian Igel. Training restricted boltzmann machines: An introduction. *Pattern Recognition*, 47:25–39, 2014.
[23] Yann LeCun. The mnist database of handwritten digits. *http://yann. lecun. com/exdb/mnist/*, 1998.
[24] Nike Dattani, Szilard Szalay, and Nick Chancellor. Pegasus: The second connectivity graph for large-scale quantum annealing hardware. *arXiv preprint arXiv:1901.07636*, 2019.
[25] Shaila Niazi et al. Training deep boltzmann networks with sparse ising machines. *arXiv preprint arXiv:2303.10728*, 2023.
[26] Andrew Rukhin et al. A statistical test suite for random and pseudorandom number generators for cryptographic applications. Technical report, 2010.
[27] Brian Sutton et al. Autonomous probabilistic coprocessing with petaflips per second. *IEEE Access*, 8:157238–157252, 2020.
[28] Shun Kanai et al. Theory of relaxation time of stochastic nanomagnets. *Physical Review B*, 103(9):094423, 2021.
[29] Keisuke Hayakawa et al. Nanosecond random telegraph noise in in-plane magnetic tunnel junctions. *Physical Review Letters*, 126(11): 117202, 2021.
[30] Christopher Safranski et al. Demonstration of nanosecond operation in stochastic magnetic tunnel junctions. *Nano Letters*, 21(5): 2040–2045, 2021.
[31] Leo Schnitzspan, Mathias Kläui, and Gerhard Jakob. Nanosecond true-random-number generation with superparamagnetic tunnel junctions: Identification of joule heating and spin-transfer-torque effects. *Phys. Rev. Appl.*, 20:024002, Aug 2023.
[32] Nihal Sanjay Singh et al. Hardware demonstration of feedforward stochastic neural networks with fast mtj-based p-bits. In *2023 International Electron Devices Meeting (IEDM) Proceedings*. IEEE, 2023.
[33] John Daniel et al. Experimental demonstration of an integrated on-chip p-bit core utilizing stochastic magnetic tunnel junctions and 2d-mos _{2} fets. *arXiv preprint arXiv:2308.10989*, 2023.
[34] Benjamin Block et al. Multi-gpu accelerated multi-spin monte carlo simulations of the 2d ising model. *Computer Physics Communications*, 181(9):1549–1556, 2010.
[35] Tobias Preis et al. Gpu accelerated monte carlo simulation of the 2d and 3d ising model. *Journal of Computational Physics*, 228(12): 4468–4477, 2009.
[36] Kun Yang et al. High performance monte carlo simulation of ising model on tpu clusters. In *Proceedings of the International Conference for High Performance Computing, Networking, Storage and Analysis*, pages 1–15, 2019.
[37] Ye Fang et al. Parallel tempering simulation of the three-dimensional edwards–anderson model with compact asynchronous multispin coding on gpu. *Computer Physics Communications*, 185(10):2467–2478, 2014.
[38] Andrea Grimaldi et al. Experimental evaluation of simulated quantum annealing with mtj-augmented p-bits. In *2022 International Electron Devices Meeting (IEDM)*, pages 22.4.1–22.4.4, 2022.
[39] K Lee et al. 1gbit high density embedded stt-mram in 28nm fdsoi technology. In *2019 IEEE International Electron Devices Meeting (IEDM)*, pages 2–2. IEEE, 2019.
